# Repurposing of the β-Lactam Antibiotic, Ceftriaxone for Neurological Disorders: A Review

**DOI:** 10.3389/fnins.2019.00236

**Published:** 2019-03-26

**Authors:** Ebrahim M. Yimer, Hailemichael Zeru Hishe, Kald Beshir Tuem

**Affiliations:** Department of Pharmacology and Toxicology, School of Pharmacy, College of Health Sciences, Mekelle University, Mekelle, Ethiopia

**Keywords:** ceftriaxone, drug repurposing, neurodegenerative disorders, Alzheimer's disease, Parkinson's disease, stroke, pain, brain ischemia

## Abstract

To date, there is no cure or disease-modifying agents available for most well-known neurological disorders. Current therapy is typically focused on relieving symptoms and supportive care in improving the quality of life of affected patients. Furthermore, the traditional *de novo* drug discovery technique is more challenging, particularly for neurological disorders. Therefore, the repurposing of existing drugs for these conditions is believed to be an efficient and dynamic approach that can substantially reduce the investments spent on drug development. Currently, there is emerging evidence that suggests the potential effect of a beta-lactam antibiotic, ceftriaxone (CEF), to alleviate the symptoms of different experimentally-induced neurological disorders: Parkinson's disease, Alzheimer's disease, amyotrophic lateral sclerosis, epileptic-seizure, brain ischemia, traumatic brain injuries, and neuropathic pain. CEF also affects the markers of oxidative status and neuroinflammation, glutamatergic systems as well as various aggregated toxic proteins involved in the pathogenesis of different neurological disorders. Moreover, it was found that CEF administration to drug dependent animal models improved the withdrawal symptoms upon drug discontinuation. Thus, this review aimed to describe the effects of CEF against multiple models of neurological illnesses, drug dependency, and withdrawal. It also emphasizes the possible mechanisms of neuroprotective actions of CEF with respective neurological maladies.

## Introduction

The conventional target-based approach for new drug discovery brings paramount challenges as well as massive economic burden, lengthy processes, and might even expose study participants to unexpected adverse events (Lee and Kim, 2016). One alternative paradigm for novel drug discovery is drug repurposing which has recently emerged as a potential strategy of off-target therapeutic actions of existing drugs (Kim, 2015; Parsons, 2018). Currently, drug repurposing is considered a promising tool for novel drug discovery as it is relatively rapid, less costly, and poses a minimal risk of adverse outcomes to study participants. These advantages would possibly overcome the challenges of the conventional *de novo* discovery of new pharmacological agents (Lee and Kim, 2016; Corsello et al., 2017).

Mental and neurological illnesses are multifarious and have a substantial influence on patients as well as being a significant economic burden for nations (Hurd et al., 2013; Gooch et al., 2017). The etiological aspects of such disorders are diverse, including pathological protein accumulation causing mostly neurodegeneration and dysregulation of the normal developmental and functional process (Di Luca et al., 2018). In 2015, neurological disorders were placed as the foremost global cause of disability and the 2^nd^ leading cause of mortality (~17% of global deaths), while Alzheimer's disease (AD) and other dementias were found to be the 4^th^ leading cause of mortality and morbidity worldwide, and is steadily among the top three causes of disability in most nations. During the period of 1990–2015, the overall mortality from neurological illnesses was increased by 37% (Feigin et al., 2017).

Moreover, there is no pharmacological agent currently available for the curative or disease-modifying actions of most of the neurological disorders. The present therapeutic approach therefore provides symptomatic management and supportive care in order to improve the longevity and quality of life of patients (Xie et al., 2014; Cummings, 2017; Dorst et al., 2018).

Thus, the repurposing of existing drugs for central nervous system (CNS) related disorders is an attractive, efficient, and dynamic drug development approach that can substantially reduce the investment spent during drug development in terms of both time and money (Caban et al., 2017; Hernandez et al., 2017).

Ceftriaxone (CEF) is a third generation cephalosporin, under the group of β-lactam antibiotics, and is the most frequently used drug for local (skin and soft tissue infections) as well as systemic community and hospital-acquired infections (Pinto Pereira et al., 2004). Recently, emerging evidence, mainly from preclinical studies, have highlighted the therapeutic efficacy of CEF against various neurological disorders, drug dependency, and withdrawal syndrome as well as its neuroprotective potential against various neurotoxic chemicals (Tables 1–3).

**Table 1:** Effects of CEF against neurodegenerative (ND) disorders.

**Models of ND disorders**	**Experimental approaches**	**Study subjects**	**Interventions**	**Major outcome (s)**	**References**
Dementia with Lewy bodies	Murine model: dementia with Lewy bodies (DLB) were induced in rats.	Male Wistar rats.	Rats were randomly divided in to the sham + saline and DLB + saline groups received saline (1 ml/kg/day) while the DLB + CEF group received CEF (100 mg/kg/day) for 27 days.	Rats that received CEF suppressed hyperactivity in the subthalamic nucleus (STN). The DLB+CEF treated group showed a significantly higher (corrected) neuronal density of neurons in the Hippocampal CA1. Rats in the DLB + CEF group also displayed a significantly lower density of α-synuclein positive cells compared to the DLB control group.	Ho et al., 2018
Parkinson's disease (PD)	Murine model: 6-hydroxydopamine (6-OHDA) induced lesion and amphetamine induced rotation in rats.	Male Sprague Dawley rats.	Rats were grouped into L-dopa alone, L-dopa + CEF, and CEF alone received groups. All groups administered unilateral 6-OHDA lesions. Saline and CEF (200 mg/kg) were administered on day 7 post-6-OHDA lesion for 7 successive days and every other week until the end of the study (day 39).	CEF meaningfully decreased abnormal uncontrolled movements at five time points inspected in the course of L-dopa treatment. Partial recovery of motor impairment from nigrostriatal lesion by L-DOPA was unaffected by CEF. CEF received L-dopa group showed a substantial increment of striatal GLT-1 expression and glutamate uptake. The loss of striatal tyrosine hydroxylase in this group was not suggestively varied compared to the L-DOPA alone received group.	Chotibut et al., 2017
	Murine model: 6-OHDA induced PD model.	Male Long Evans rats.	In Experiment 1, a 100 mg/kg of CEF for nine rats and, in Experiment 2, the 50 mg/kg CEF alone and along with L-DOPA for 16 rats were administered. Saline and L-DOPA separately were uses as negative and positive controls, respectively.	Daily administration of CEF (100 mg/kg) significantly enhanced the contralateral forepaw stepping (~44%) (this action continued for 1 month after CEF discontinuation). The daily administration of 50 mg/kg CEF showed comparable efficacy to 10 mg/kg of L-DOPA in amplifying the contralateral forepaw stepping (~40%). Moreover, CEF did not produce dyskinesia instead reduced its development but did not affect the occurrence of L-DOPA induced dyskinesia.	Kelsey and Neville, 2014
	Murine model: 6-OHDA lesioned PD model.	Male Sprague–Dawley rats.	For naive (not 6- OHDA-lesioned) rats, CEF was injected (200 mg/kg), while the control group administered saline for 7 subsequent days. In animals underwent 6-OHDA lesion, CEF was administered on the same day of lesion and for subsequent 1 week. Whereas, a control group were administered only a vehicle.	The 200 mg/kg CEF injection showed an elevation of striatal glutamate uptake in non-lesioned rats and this effect persisted till 2 weeks post-injection CEF administration in 6- OHDA lesioned rats showed a substantial attenuation (~57%) of tyrosine hydroxylase (TH) loss compared to corresponding vehicle-received group (~85%) CEF also decreased amphetamine-induced rotation and locomotor behavior alteration induced by 6-OHDA This reduction of TH loss was accompanied with augmented glutamate uptake and GLT-1 expression.	Chotibut et al., 2014
	Murine model: 6-OHDA lesioned PD model.	Male Sprague–Dawley rats.	For naive (not 6- OHDA-lesioned) rats, CEF was injected (200 mg/kg), while the control group administered saline for 7 subsequent days. In animals underwent 6-OHDA lesion, CEF was administered on the same day of lesion and for subsequent 1 week. Whereas, a control group were administered only a vehicle.	The 200 mg/kg CEF injection showed an elevation of striatal glutamate uptake in non-lesioned rats and this effect persisted till 2 weeks post-injection. CEF administration in 6- OHDA lesioned rats showed a substantial attenuation (~57%) of tyrosine hydroxylase (TH) loss compared to corresponding vehicle-received group (~85%). CEF also decreased amphetamine-induced rotation and locomotor behavior alteration induced by 6-OHDA. This reduction of TH loss was accompanied with augmented glutamate uptake and GLT-1 expression.	Chotibut et al., 2014
	Murine model: MPTP induced models of PD in rats.	Male Wistar rats.	Rats were randomly grouped into 7 and all groups except vehicle-treated group injected MPTP repeatedly. Group-I: Sham control group, Group-II: saline, Group-III: CEF (100 mg/kg), Group-IV: CEF (200 mg/kg), Group-V: Ropinirole (1.5 mg/kg), Group-VI: Ropinirole (3 mg/kg), Group-VII: CEF (100 mg/kg) + Ropinirole (1.5 mg/kg).	CEF (100 and 200 mg/kg) received animals meaningfully enhanced the motor impairments. CEF injection diminished the oxidative injury and restored the reduced level of endogenous antioxidant enzymes. CEF also meaning fully attenuated the pro-inflammatory cytokines such as TNF-α and IL-β in striatum region. Ropinirole pre-treatment with lower dosage of CEF remarkably enhanced the protective effect of CEF as compare to CEF alone.	Bisht et al., 2014
	Murine model: MPTP-induced PD rat model.	Male Wistar rats.	The MPTP-injected group received either CEF (200 mg/kg/day) (CEF group; *n =* 11) or saline (saline group; *n =* 14), while the sham-operated group received saline injections but not MPTP (saline group; *n =* 12).	CEF attenuated the MPTP-induced memory impairments. CEF also averted the MPTP lesion-induced degeneration of DAergic neurons in the nigrostriatal area. It also terminated the microglial activation in the substantia nigra pars compacta (SNc) and cellular loss in the hippocampal CA1 region.	Ho et al., 2014
	Murine model: MPTP-induced PD rat model.	Male Wistar rats.	CEF or saline was injected for 15 subsequent days with the regimes of: the sham-operated group injected with saline (1 ml/kg/day, *n =* 13), while the MPTP-lesioned rats were subdivided into either saline (1 ml/kg/day, *n* = 13), or CEF (5 mg/kg/day, *n =* 13) groups.	MPTP lesioning-induced impairment of motor function, working memory, and object recognition were prevented by CEF. Administration of CEF also ameliorated behavioral deficits in MPTB-induced PD rat's model. CEF also prevented MPTP-induced neuronal loss and partly promote neurogenesis.	Hsieh et al., 2017
	Murine model: MPTP-induced PD rat model.	Male Wistar rats.	The sham-operated groups injected with either saline (*n =* 10) or CEF at a dose of 100 (*n =* 11) or 200 mg/kg/day (*n =* 12), while the MPTP-lesioned groups received saline (*n =* 11) or CEF at the dose of 100 or 200 mg/kg daily beginning either on day 5 or day 3.	Rats that received either pre- or post-lesioning with CEF prevented the DAergic degeneration in SNc and striatum and improved working memory and object recognition. Lesioning that could cause neurodegeneration and glutamatergic hyperactivity were markedly suppressed by CEF treatment. CEF received group also displayed an upregulated GLT-1 expression.	Hsu et al., 2015
	Murine model: MPTP-induced Parkinson's disease (PD) rat model.	Male Wistar rats.	After MPTP lesioning, animals were injected with daily CEF (5 mg/kg), erythropoietin (100 IU/kg), or CEF + erythropoietin (EPO) and undertook the bar-test, *T*-maze test, and object recognition test. Saline was given both for sham-operated and one of MPTP received groups as control.	Memory impairments were prominently decreased or abolished in rats administered either CEF alone or in combination with EPO. The co-administration of the 2 agents (CEF + EPO) showed superior action than individual agents. CEF, EPO, or CEF + EPO also reduced or eliminated MPTP lesioning-induced neurodegeneration. The combined administration of these agents also displayed a better outcome in the densities of DAergic terminals.	Huang et al., 2015
	Murine model: MPTP-induced PD models in rats.	Male Wistar rats.	In experiment 1 (5 groups, each consisted of 7 rats), CEF (100 and 200 mg/kg) was administered for rats after 14th day of MPTP injection for 2 weeks. In experiment 2 (4 groups, each consisted of 7 rats), CEF (100 mg/kg) and Memantine (20 mg/kg) were initiated after 15th day of Dihydrokainate administration in MPTP received groups.	CEF (200 mg/kg) treated rats exhibited substantial betterment of behavioral impairment and oxidative damage. CEF also attenuated the marked upsurge of neuroinflammatory markers including NFκB, TNF-α, and IL-1β in MPTP received groups. CEF significantly recovered the reduced activity of BDNF in MPTP received rats. Pre-treatment of memantine with lower dose of CEF enhanced the protective actions of CEF.	Kaur and Prakash, 2017
	Murine model: 6-OHDA lesion-induced PD model.	Sprague–Dawley rats.	The treatment groups were received CEF (200 mg/kg/day) for 1 week prior to lesion surgery, while the control groups were administered a 0.9% saline for the same duration.	CEF pre-treatment ameliorated the muscular rigidity and contralateral rotation in 6-HODA-lesioned rats. CEF treated group also showed a reduction of dopaminergic neuronal deaths. Both protein expression and TH immunoreactivity for GLT-1 were overexpressed in CEF treated rats.	Leung et al., 2012
	*In vitro* study: 6-OHDA exposed PC12 cells.	Neural (PC12) cells.	PC12 cells were treated with varying concentrations of CEF (10–100 μM) alone or along with a 6-OHDA for 24 h. For separate experiment, 100 thousand PC12 cells/cm^2^ were plated and treated after 1 day.	CEF interacted with high affinity to α-synuclein and interfered with the *in vitro* polymerization. PC12 cells pre-treatment with CEF (100 μM) resulted in a down-regulation of α-synuclein expression, but not at 10 μM concentration). CEF at the concentration of 100 μM, showed a substantial recovery of cell apoptosis and restore PC12 viability. Both lower (10 μM) and maximal conc. (~1 mM) failed to recover 6-OHDA-induced apoptosis.	Ruzza et al., 2014
	Murine model: MPTP-induced PD rat model.	Male Wistar rats.	Rats were grouped (5 rats/group) into sham-operated and two MPTP-lesioned groups; the sham group received saline, while the MPTT-lesioned groups received either daily saline or CEF (100 mg/kg) for 15 days.	CEF treatment prevented the MPTP-induced decreases in neurogenesis of rats in the dentate gyrus of the hippocampus. CEF treatment also ameliorated the decrease in both neuronal density and activity in the nigrostriatal, hippocampus, and subthalamic nucleus observed in the MPTP-induced rats. Besides, unlike saline received group, CEF treated group showed an intact density of DAergic neurons in the SNc and DAergic terminals in the striatum.	Weng et al., 2016
	*In vitro* study: astrocytes exposed to a neurotoxin, 1-methyl-4- phenyl-pyridinium (MPP^+^) model.	Cultured primary astrocytes from Sprague–Dawley rat pups.	After cells were cultured, they were randomly divided into (a) control group (received saline); (b) MPP^+^ treatment group (received culture medium having MPP^+^); (c) CEF treatment group (received culture medium having 100 μM CEF); and (d) Co-treatment with MPP^+^ and CEF group (received culture medium containing MPP^+^ plus 100 μM CEF).	CEF (100 μM) enhanced the expression and uptake of glutamate in astrocytic cells exposed to MPP+. CEF also upregulated the expression of GLT-1 and stimulates astrocyte viability after *in vitro* MPP^+^ exposure. CEF Attenuated MPP^+^-induced apoptosis, cytotoxicity and neurotoxicity in primary astrocytes. The cytoprotective actions of CEF is mediated through downregulation of NF-κB and JNK/c-Jun Signaling.	Zhang et al., 2015
Alzheimer' s disease (AD).	*In vitro* and Murine model: triple trans- genic model of AD [3xTg-AD].	3xTg-AD mice; astrocyte and neuron 1^0^ cell culture.	Mice were grouped in to CEF and vehicle treated group: 4 female and 4 male mice received saline (control group) while 3 female and 4 male mice received CEF (200 mg/kg).	Prolonged administration of CEF in aged 3xTg-AD mice showed a substantial upregulation GLT-1 expression, improving cognitive impairments, preserving synaptic proteins and reducing of tau pathology. But both beta amyloid (Aβ) and amyloid precursor protein were not significantly altered in CEF treated group compared to control group.	Zumkehr et al., 2015
	Murine model: rat model of accelerated senescence.	Male Wistar and OXYS rats.	Animals were administered daily with either saline (Wistar + saline, *n =* 10 and OXYS + saline groups, *n =* 10) or CEF at the dose of 50 (OXYS + CEF50 group, *n =* 10) or 100 mg/kg (OXYS + CEF100 group, *n =* 10) for 36 days.	Long-term treatment of CEF in a dose of 100 mg/kg moderately improved movement impairments and recovered the deficit of new object recognition. Both doses of CEF (50 and 100 mg/kg) increased the density of pyramidal neurons in the CA1 area in OXYS rats. A 50 mg/kg doses of CEF also expressively amplified the immunoreactivity of TH in the striatum.	Tikhonova et al., 2017
	Genetic murine model: on two different genotypes rats genotype.	Male Wistar rats, OXYS male rats.	Beginning from the age of 14 weeks, animals were administered either daily CEF (100 mg/kg) or saline for 36 days.	CEF treated group showed downregulation of mRNA levels of *Bace1* (that encodes β-secretase involved in Aβ production) and *Ace2* (that encodes enzymes involved in Aβ degradation) in the hypothalamus. CEF received group also exhibited diminution of the expression of gene of β-actin (*Aktb*) in the frontal cortex. CEF amplified *Mme, Ide* (that encode enzymes involved in Aβ degradation). CEF also upregulated the expression of *Epo* mRNA level in the amygdala and the levels of *Ece1* and Aktb in the striatum.	Tikhonova et al., 2018
	Murine model: scopolamine-induced memory impairment in mice.	Swiss albino mice.	Animals were divided in to 6 groups (6 mice/group): group I (vehicle), group II (vehicle + scopolamine), group III (Donepezil + scopolamine), group IV (206 mg/kg of CEF + scopolamine), group V (0.49 mg/kg of selegiline + scopolamine), group VI (206 mg/kg of CEF + 0.49 mg/kg of selegiline + scopolamine).	CEF and selegiline improved scopolamine induced cognitive impairment. Both drugs (CEF and selegiline) showed a substantial memory enhancing ability compared to negative control. Elevated level of acetylcholine esterase (AchE) were attenuated in mice that received either CEF or selegiline. CEF or selegiline received mice also showed improved oxidative status. Concomitant administration of CEF and selegiline displayed a synergistic action on cognitive-improvement, AchE and antioxidant activities.	Akina et al., 2013
	Murine model: APP/PS1 transgenic AD model.	PP/PS1 transgenic AD mice model.	Animals were assigned: wild type (received normal saline), APP/PS1 (saline) and CEF (100, 200, and 300 mg/Kg) groups. All these groups of the APP/PS1 mice were treated with either the vehicle or CEF once daily for 2 weeks. One additional group was also received 200 mg/Kg CEF after receiving dihydrokainate.	CEF received group substantially enhanced the cognitive impairment in early stage of AD animals. CEF (all the 3 doses) also upregulated GLT-1, glutamine synthetase (GS), and system N-glutamine transporter-1 (SN1) protein expressions in the hippocampus of APP/PS1 AD mice. Dihydrokainate, a selective inhibitor of GLT-1 reversed enhanced memory functioning, GS activity, and SN1 expression of CEF in APP/PS1 AD mice.	Fan et al., 2018
	Murine model: APP/PS1 AD model.	Mice of either APP/PS1 or wild-type mice.	Mice were allocated into CEF treated (APP/PS1 and wild-type mice: 200 mg/Kg CEF for 5 days, *n =* 9) and control groups (APP/PS1 and wild-type mice received vehicle for five subsequent days, *n =* 5/group).	CEF administered group showed partial restoration of the reduced GLT-1 level in the area around to Aβ plaques. CEF treated mice also displayed a marked decrement of the chronically overexpressed glutamate levels that were identified in the vicinity of the amyloid aggregate. Hence the neurotoxic microenvironment encompassing Aβ aggregates were substantially improved in CEF received group of mice.	Hefendehl et al., 2016
Amyotrophic lateral sclerosis (ALS).	A multi-phase randomized trial of human-subjects diagnosed with ALS.	Sixty-six human subjects having defined ALS.	In Stage 1, participants at 10 clinical sites were randomized into 3 equal study groups receiving either daily placebo or CEF (2 gm or 4 gm in to 2 divided doses). Study groups were continued their allocated treatment in Stage 2.	The stage 1 investigation showed a linear PK, and CSF trough levels for both low and high dose levels beyond the pre-stated target trough level. Tolerability outcomes (Stages 1 and 2) displayed that CEF at dosages up to 4 gm/day was well-tolerated until 20 weeks. Biliary related side effects were more common with CEF, which were dose independent and were overcome with ursodeoxycholic treatment.	Berry et al., 2013
	A multi-stage, randomized, double-blind, placebo-controlled trial.	Five hundred and fourteen eligible adult patients with ALS.	In stages 1 (PK) and 2 (safety), participants were randomly assigned to daily CEF (2 g or 4 g) or placebo. In stage 3 (efficacy), participants allocated to CEF in stage 2 received 4 g CEF and new participants were randomly assigned to 4 g CEF or placebo. To reduce biliary adverse events of CEF, CEF received group also administered ursodeoxycholic acid (300 mg BID).	In the period of stages 1 and 2, mean ALSFRS-R scores were decreased gradually in participants who administered CEF (4 gm) compared to placebo. Nonetheless in stage 3 both functional decline and survival between groups were not substantially differed. Handheld dynamometry endpoints in patients who completed the study displayed no noticeable differences between groups. GIT and hepatobiliary associated adverse effects were more common in CEF groups than control group.	Cudkowicz et al., 2014
	*in vitro* study: from tissue culture.	Mouse embryonic fibroblast, HT22 cell, human motor neurons derived from embryonic stem cells.	Cells were treated either with CEF (30, 100 or 300 μM) or vehicles.	Chronic CEF treatment prevent all tested cells against oxidative glutamate toxicity and induces EAAT expression in dose dependent fashion. CEF-mediated protection is related with upregulation of xCT expression, amplified activity of system xC^−^ (↑se GSH). CEF-induced amplification of glutathione (GSH) level was related with an increase in system ${\text{x}}_{\text{C}}^{-}$ activity). CEF exhibited a slow upregulation of Nrf2 without any pro- or antioxidant activity.	Lewerenz et al., 2009
	Surgical model: axotomized mice model.	Adult male C57BL/6 mice were used.	Twenty-six axotomized mice were grouped into either drugs (CEF or minocycline) or saline-received groups from the 1st day of hypoglossal axotomy: CEF-administered group (200 mg/kg, *n =* 8), minocycline-received group (30 mg/kg, *n =* 8), and vehicle- administered group (5 ml/kg, *n =* 10).	Both CEF and minocycline treatment group showed a substantial enhancement of survival rate of lesioned motor neurons. But there are no noticeable differences in the cellular densities of astrocytes in CEF treated and control groups. CEF upregulated the expression of GLT-1 in the hypoglossal nucleus, while it inhibited the reactive increment of the glial protein expression.	Yamada and Jinno, 2011
Huntington's disease (HD).	Murine model: R6/2 mice, transgenic model of HD.	Male transgenic R6/2 mice (HD phenotype).	Animals were treated with either CEF (200 mg/kg), or same volume of vehicle (saline) once daily for 5 subsequent days.	CEF treatment in R6/2 mice attenuated various manifestation of HD including a decreased paw clasping and twitching, while motor flexibility and open-field climbing were amplified. Compared to vehicle received group, CEF treated group showed an upregulation of GLT1 expression in striatum. CEF also restored the glutamate uptake in striatum of R6/2 mice. Upregulation of the functional GLT1 level by CEF attenuated the multiple HD behavioral phenotype.	Miller et al., 2008
	Murine model: R6/2 model of HD in mice.	Male transgenic R6/2 and Wild type mice.	Mice were administered either daily CEF (200 mg/kg) or vehicle for 5 successive days.	CEF treatment upregulated the expression of cortical and striatal GLT1 level compared to saline treated group. This action is persisted even after GLT1 levels began to decrease when these mice are 13 weeks of age and overtly symptomatic. Therefore, the cellular machinery underlying the CEF- upregulated GLT1 expression possibly working in late-stage HD.	Sari et al., 2010

*MPTP, 1-methyl-4phenyl-1,2,3,6 tetrahydropyridine; 6-HODA, 6-Hydroxydopamine; CEF, Ceftriaxone; ALS, Amyotrophic Lateral Sclerosis; ALSFRS-R, Amyotrophic Lateral Sclerosis Functional Rating Scale-Revised; GLT 1, glutamate transporter 1; EAAT2, excitatory amino acid transporter subtype 2; HD, Huntington's disease; PD, Parkinson's disease; CSF, Cerebrospinal fluid; PK; pharmacokinetic; AD, Alzheimer's disease**;** AchE, Acetylcholine esterase*.

**Table 2:** Effect of CEF against ischemia, pain, traumatic brain injury, and stroke.

**Model of disorders**	**Experimental approach**	**Study subjects**	**Interventions**	**Major treatment outcomes**	**References**
Ischemia	Murine model: hypoxic-injury (H-I) induced ischemia in rats.	Male and female Sprague Dawley rats.	Animals were randomly divided and pretreated with CEF (200 mg/kg), minocycline (45 m/kg), erythromycin (25 mg/kg) and equal volume of saline for 5 subsequent days. Then, rats underwent hypoxic-ischemic (H-I) or shame operated procedure.	CEF treatment meaningfully increased the expression of GLT-1 mRNA and protein levels; CEF pretreated animals showed a reduction of infarct size and apoptotic index. CEF treated animals also showed increment in microtubule-associated protein 2 -positive area, while decreased the TUNEL-positive cells.	Mimura et al., 2011
	Murine model: focal ischemic cortical lesions in rats.	Male Long-Evans hooded rats.	Animals were allocated to receive CEF at a dose of 200 mg/kg (*n =* 12) or vehicle (*n =* 11) daily for 5 days. Then, the effect of CEF on motor skill learning and rehabilitative training-induced functional enhancement after ischemic lesions was assessed.	In normal animals, CEF failed to influence the skill learning rate or final level of reaching performance. After rats underwent ischemic lesion, CEF exacerbated initial deficits in reaching performance. CEF also affected acquisition of the rehabilitative training task. There is no significant difference in lesion size between CEF and vehicle received groups.	Kim and Jones, 2013
	Murine model: cortical vein occlusion by photochemical thrombosis in rats.	Male Wistar rats.	Animals were assigned to receive CEF (100 or 200 mg/kg), vehicle or vehicle or CEF together with GLT 1 inhibitor, Dihydrokainate (DHK) for 5 days prior to venous ischemia.	CEF treated animals showed to decrease infarct volume compared to vehicle received group. The effect of CEF was reduced by administration of the GLT-1 inhibitor, DHK. This suggests that GLT-1 is essential for the observed actions of CEF; the effect of CFT on NTs receptor density did not significantly differ from vehicle treated rats.	Inui et al., 2013
	Murine model: rat model of global brain ischemia (GBI).	Male Wistar rats.	The animals were randomly assigned to sham group, CEF group (50, 100, and 200 mg/kg). The CEF was administered as control, pre- and post-treatment groups.	CEF pre-treatment expressively prevented delayed neuronal death of pyramidal neurons in hippocampal CA1 area induced by GBI in dose-dependent fashion. Pre-administration with CEF also up-regulated the expression and glutamate uptake of GLT-1. Inhibitors of GLT-1, DHK significantly reduced CEF-induced up-regulation of GLT-1 and its neuroprotective effect against global ischemia.	Hu et al., 2015
	Murine model: global brain ischemia/ reperfusion (I/R) injury in rats.	Wistar-albino rats.	Animals were allocated in to control (*n =* 10), I/R (*n =* 10), and I/R-CEF (*n =* 10, 100 mg/kg CEF 2 h before I/R) groups.	The levels of MDA was meaningfully decreased, while increased the activity of SOD and GSH in the I/R-CEF group compared with the I/R and control groups. CEF provided morphological improvement of histopathological (microvessel and neuron structure) analysis of brain tissue than the I/R group; there were no significant differences observed in the level of NO among the groups.	Altaş et al., 2013
	Murine model: focal cerebral ischemia in rats.	Male Sprague Dawley rats.	Animals were randomly allocated to sham group (*n =* 12), control group (*n =* 30), and CEF (200 mg/kg) pretreated group (*n =* 30) for 5 days.	CEF pre-treatment improved the scores of neurological deficit. It also significantly decreased the percentage of infarct volumes mainly in the cortex but not in the striatum. After reperfusion, the activation of microglia cells was decreased and the expression of IL-1β was partially inhibited in rats that received CEF than control group.	Lujia et al., 2014
	Murine model: focal cerebral ischemia in rats.	Male Wistar rats.	Animals were assigned to receive CEF (200 mg/kg), n-acetyl cysteine (NAC) (150 mg/kg), or saline for 5 subsequent days.	CEF meaningfully reduced infarct size and improved neurological deficits induced by a middle artery cerebral occlusion. CEF treated group showed an enhanced level of GLT 1 expression in the dorsal striatum. In ischemic rats, CEF also upregulated the expression of xC- mRNA in frontal cortex and dorsal striatum. CEF not NAC treated group also upregulate the GLT-1 expression in the frontal cortex and dorsal striatum.	Krzyzanowska et al., 2016
	Murine model: neonatal rat model of hypoxic-ischemic encephalopathy (HIE).	Neonatal Sprague Dawley (SD) rats.	Neonatal rats were administered either of CEF (50, 100, 200 mg/kg,) or saline 2 days before experimental HIE.	Pre-treatment with CEF (200 mg/kg) substantially attenuated the scores of neonatal rats' brain injury. CEF treated group also decreased the extent of apoptotic cells in the hippocampus. Animals that received CEF restored myelination and improved learning and memory deficit induced by HIE. CEF treated group also showed an upregulation of GLT1 expression in the cortical neurons.	Lai et al., 2011
Pain	Murine model: chronic constrictive nerve injury (CCI) in rats.	Male Sprague Dawley rats.	Rats were allocated to receive vehicle or CEF (200 mg/kg) the CEF group were ether administered with CEF (200 mg/kg) once daily for 7 days beginning immediately after CCI starting on postoperative day (day 9).	Both preventive and therapeutic CEF administration showed an up-regulation of GLT-1 expression and glutamate uptake; the action was suppressed by GLT-1 blocker. Pre-treatment of CEF prevented the development of mechanical allodynia and thermal hyperalgesia induced by CCI (anti-nociceptive effects). Therapeutic administration of CEF also significantly increased the latency of thermal withdrawal and the threshold of mechanical withdrawal. The preventive or therapeutic injection of CEF displayed an inhibiting effect on the development and maintenance of mechanical allodynia.	Hu et al., 2010
	Murine model: opioid-induced hyperalgesia (OIH) in mice.	Male ICR mice.	Mice received morphine sulphate (20 mg/kg) BID for 3 days and two more injections of 40 mg/kg morphine sulfate on 4th day. Mice were received CEF (200 mg/kg) before morphine injections and continued for 1 week.	CEF administration exhibited prevention of OIH behavior. It also alleviated the mechanical allodynia, and thermal hyperalgesia induced by repeated administration of opioid. CEF also recovered the downregulation of GLT-1 expression brought by OIH.	Chen et al., 2012
	Murine model: spinal nerve ligation induced neuropathic pain in rats.	Male SD rats.	Animals were allocated to receive control, pioglitazone (5, 10, or 20 mg/kg), CEF (100 or 200 mg/kg) alone or combination for 28 days.	CEF (200 mg/kg) administration substantially improved the paw withdrawal threshold. Treatment with CEF monotherapy (200 mg/kg) expressively prevented the behavioral, biochemical, mitochondrial and cellular alterations. CEF (200 mg/ kg) also significantly attenuated the TNF-a, IL-6 and caspase-3 activities.	Pottabathini et al., 2016
	Murine model: chronic constriction injury model (CCI) in rats.	Male Wistar rats.	Animals were assigned to receive control, CEF (100, 150, and 200 mg/kg) or minocycline (25, 50, and 100 mg/kg) alone or combination of CEF and minocycline for 1 week.	CEF produced a dose-dependent reversal effects of the neuropathic pain behaviors. Administration of CEF (200 mg/kg) significantly attenuated CCI induced production of TNF-α and IL-1β than control group. The highest dose of CEF (200 mg/kg) attenuated tactile allodynia in CCI rats.	Amin et al., 2012
	Murine model: somatic Inflammatory Hyperalgesia in rats and Visceral Nociception in mice.	Male Wistar rats and Swiss Webster mice.	Animals were allocated to control, CEF (10–200 mg/kg) alone and in combinations with various analgesic drugs in carrageenan-induced paw inflammatory hyperalgesia and in the acetic acid-induced writhing test for 7 days.	Pre-treatment with CEF for 1 week showed a significant analgesic action in the somatic inflammatory model in dose-dependent manner. Pre-administration of CEF also exhibited a substantial antinociceptive effect in the visceral pain model in dose-dependent fashion. Combination of CEF and different analgesics showed a synergistic reduction of hyperalgesia and nociception in both somatic and visceral inflammatory pain than separated drugs used.	Stepanovic-Petrovic' et al., 2014
	Murine model: chronic constriction injury (CCI) in rats.	Male Wistar rats.	Animals were randomly allocated into saline, sham-operated and CEF (200 mg/kg) for 1 week.	CCI rats that received CEF showed a remarkable upregulation in the levels of antiapoptotic protein, Bcl2, and decreased the contents of Bax protein. CEF also attenuated the cleaved forms of caspases 3 and 9. CEF treated groups were also protected from the CCI-brought oxidative stress.	Amin et al., 2014
	Murine model: mechanical allodynia and hyperalgesia through STZ induced neuropathic pain.	Male Wistar rats.	Rats were injected either daily CEF (50–200 mg/kg) or control for 1 week.	CEF increased in the paw withdrawal thresholds. The GLT- 1 transporter inhibitor, DKA, recovered anti-allodynic and anti-hyperalgesia actions of CEF. The highest dose of CEF (200 mg/kg) significantly reduced both mechanical allodynia and hyperalgesia.	Gunduz et al., 2011a
	Murine model: radicular pain through dorsal nerve root compression in rats.	Male Holzman rats.	Animals were randomly allocated to receive either saline or CEF (40 μl of 10 μg intrathecal injection).	CEF treatment also attenuated both mechanical allodynia and modulated the expression of GLAST and GFAP. CEF injection also restored the reduced spinal astrocytic activity, and neuronal hyperexcitation in the spinal dorsal horn. CEF administration showed a significant upregulation of GLT 1 expression.	Nicholson et al., 2014
	Murine model: peripheral pain induced by injections of formalin; neuropathic pain using the spinal nerve ligation (SNL).	Male Sprague Dawley rats.	Animals were received either CEF (200 mg/kg) or same volume of saline.	CEF significantly increased both mechanical and thermal withdrawal threshold in naïve rats. After induction of neuropathic pain by SNL, effects of CEF was substantial in reducing thermal hyperalgesia but failed to show significant effect on mechanical allodynia. CEF also delayed the intensity of painful behaviors in response to formalin induced inflammatory hyperalgesia.	Eljaja et al., 2011
	Murine model: experimental autoimmune encephalomyelitis (EAE) and chronic constriction nerve injury (CCI) models.	Male Sprague Dawley rats and male Dark Agouti rats.	Animals were received either saline or CEF (150 μg intrathecal).	Pre-administration of CEF attenuated the development of hyperalgesia and allodynia in response to repeated morphine. In experimental EAE, CEF displayed tactile allodynia and arrested the progression of motor weakness and paralysis. Similarly, CEF recovered tactile allodynia induced by CCI. CEF treatment also reversed the EAE induced downregulation of GLT-1 and glial activation. CEF also restored CCI- and EAE induced astrocytic activation in lumbar spinal cord.	Ramos et al., 2010
	Murine model: chronic constriction nerve injury (CCI) in rats.	Male Wistar rats.	Rats were allocated to receive CEF (100, 200 or 400 mg/kg), clavulanic acid (CLA) (0.1, 1, or 10 mg/kg) gabapentin (10 mg/kg as positive control) or saline.	Both CEF and CLA produced antiallodynic effects in response to mechanical and cold stimulation in dose dependent fashion. The antiallodynic effect of CEF was antagonized by haloperidol and naloxone, which indicates CEF may be mediate its antiallodynic actions through dopamine and opioid receptors. Moreover, the plasma TNF-α levels were attenuated following the highest dose of CEF (200 mg/kg) and CLA (10 mg/kg) treatment.	Ochoa-Aguilar et al., 2017
	Double-blind randomized trials and Murine model.	Forty five human subjects and male C57/ Black mice.	Patients were randomly allocated to receive an IV infusion of saline (*n =* 15), CEF (2 gm) (*n =* 15), or cefazolin (2 g) (*n =* 15) 1 h prior to surgery. Similarly, in the animal model of postoperative pain, mice were assigned to receive a single dose of saline, CEF (200 mg/kg), or cefazolin (200 mg/kg).	A single dose CEF produced a substantial analgesia in all patients. The whole patients responded to CEF and about 10-fold increment in the threshold of nociception. A single injection of CEF exhibited a substantial antinociceptive effects in mouse models of inflammatory or postsurgical pain. CEF also increased the expression of GLT-1 (2-fold) in the spinal cord.	Macaluso et al., 2013
Traumatic Brain Injury (TBI).	Murine model: rat model of TBI.	Male Sprague Dawley rats.	Animals were randomly assigned to sham group (*n =* 30), TBI group (*n =* 60) and TBI treated CEF group (*n =* 60, 200 mg/kg). CEF was injected for 5 days starting just after TBI and both sham and TBI groups administered equal volumes of saline.	Daily administration of CEF attenuated TBI-induced brain edema and cognitive impairments in rats. CEF treated group restored TBI induced downregulation of GLT-1 expression. CEF also revealed a substantial suppression of autophagy marker protein, LC3 II, in hippocampus than the TBI group.	Cui et al., 2014
	Murine model: rat lateral fluid percussion injury TBI model.	Male Long-Evans rats.	Animals were divided in to “saline-sham,” “saline-TBI,” and “CEF-TBI” (CEF, 200 mg/kg) groups and treatment continued daily for 7 days after TBI surgery.	CEF injection reduced the level of regional glial fibrillary acid protein (GFAP) expression (43%) in the lesioned cortex. CEF received group also showed a notable attenuation of cumulative post-traumatic seizure duration. The reduced level of GLT-1 expression after TBI was restored by administration of CEF.	Goodrich et al., 2013
	Murine model: TBI model in rats.	Sprague Dawley rats.	Rats were randomly allocated to sham-operated group (*n =* 18), trauma group (TBI, *n =* 27) and trauma + CEF treatment group (TBI + CEF, *n =* 27). After TBI, CEF (200 mg/kg) was administered in TBI + CEF group just after trauma, while both sham and TBI groups received only vehicle.	CEF treated group showed an attenuation of TBI-induced cerebral edema and functional cognitive impairments. CEF administration also decreased the levels of proinflammatory cytokines interleukin-1β, interferon-γ, and TNF-α. It also up-regulated the expression of GLT-1 level after TBI.	Wei et al., 2012
	*in vitro* and Murine model: a rat model of subarachnoid hemorrhage (SAH).	Male Sprague Dawley (SD) rats and Primary astrocyte cultures.	Animals were assigned randomly to sham, CEF (SAH + 50 or 100 mg/kg) and vehicle (SAH + saline) groups.	CEF meaningfully alleviated the SAH-induced cognitive deficits in spatial learning memory and reference memory. CEF administered rats showed a reduction of hippocampal neuronal apoptosis following SAH. CEF treated animals also reversed the downregulation of EAAT2 expression expressively following SAH. CEF also enhanced the nuclear translocation of p65 and the activation of Akt in hippocampal astrocytes.	Feng et al., 2014
Stroke	Murine model: experimental stroke through middle cerebral artery occlusion in rats.	Male normotensive Wistar rats.	Rats were randomly allocated to receive vehicle (sham, *n =* 8), CEF (sham, *n* = 13; CEF, 200 mg/kg), vehicle [stroke, *n =* 29, CEF (stroke, *n =* 19; 200 mg/kg)].	CEF received group displayed a substantial reduction of early mortality (from 34.5 to 0%). CEF treatment also meaningfully decreased the infarct size and improved neuronal survival within the penumbra. CEF further ameliorated neurological dysfunctions, which led to an overexpression of neurotrophins in the peri-infarct area and also upregulated the expression of GLT1 level.	Thöne-Reineke et al., 2008
	*In vitro* model of stroke.	Hippocampal slices obtained from adult rats and orga- notypic hippocampal cultures.	Acute hippocampal slices obtained from rats and organotypic hippocampal slices treated with either vehicle or CEF for 5 days (200 mg/kg.) and exposed to oxygen-glucose deprivation.	CEF treated group delayed the occurrence of oxygen-glucose deprivation-induced hypoxic spreading depression (neuroprotective effect). The glutamate-induced NMDA currents from CA1 pyramidal neurons showed a greater enhancement of these currents in CEF-treated groups. But pre-treatment of slice cultures with CEF (10–200 μM for 5 days) failed to affect either the 1^0^ (direct) or 2^0^ (delayed) damage of CA1 pyramidal neurons brought by glutamate and CEF exposure also did not increase GLT-1 expression.	Lipski et al., 2007

*CCI, Chronic constriction nerve injury; CEF, Ceftriaxone; GLT-1, Glutamate transporter 1; H-I, Hypoxic-injury; I/R, Ischemia/reperfusion; SAH, subarachnoid hemorrhage; TBI, Traumatic brain injury; HIE, Hypoxic-ischemic encephalopathy; TUNEL, Transferase-mediated deoxy-uridine triphosphate nick end labelling; SNL, Spinal nerve ligation; EAE, experimental autoimmune encephalomyelitis; TNF-α, Tumor necrosis factor-alpha; OIH, Opioid-induced hyperalgesia*.

**Table 3:** Effect of CEF on different types drug/alcohol of dependencies and withdrawals.

**Type of dependence/ Withdrawal**	**Model Used**	**Animals used (species, sample)**	**Intervention**	**Main outcome (s) upon CEF administration**	**References**
Ethanol dependence	Murine model: animal model of alcoholism	Alcohol-preferring (P) rats	25, 50, 100, or 200 mg/kg CEF (CEF, i.p.)	Significant reduction in daily ethanol consumption. Dose-dependent increases in water intake. Body Weight did not affect. Not reduction in sucrose. Increases in GTL1 expression levels within the PFC and NAc with the large dose.	Sari et al., 2011
	Murine model: animal model of alcoholism.	Alcohol-preferring (P) rats.	100 mg/kg CEF (i.p.).	Significant reduction in daily ethanol consumption. Dose-dependent increases in water intake. Body Weight did not affect. Increased GLT1 level in the NAc and its shell. Down regulated ENT1 in the NAc and its shell.	Sari et al., 2013
	Murine model: animal model of alcoholism.	Alcohol-preferring (P) rats.	100 mg/kg CEF (CEF, i.p.).	Significant reduction in daily ethanol consumption. Dose-dependent increases in water intake. Body Weight did not affect. Increases in GTL1 expression levels within the PFC and NAc with the large dose.	Rao and Sari, 2014a,b
Ethanol withdrawal.	Murine model: model of ethanol withdrawal.	Adult male P rats and Wistar rats.	CEF (50 or 200 mg/kg; i.p.).	Completely abolished all manifestations of ethanol withdrawal. Prevented withdrawal-induced escalation of alcohol intake. Upregulation of EAAT 2 in the striatum.	Abulseoud et al., 2014
	Murine model: animal model of alcohol dependence.	Alcohol-preferring (P) rats.	CEF (50 or 100 mg/kg) was administered.	CEF treatment attenuates relapse-like ethanol-drinking behavior. Showed a reduction in ethanol intake compared to control group. Upregulation of GLT1 level in prefrontal cortex and NAc. CEF has no effect on relapse-like sucrose-drinking behavior.	Qrunfleh et al., 2013
Nicotine withdrawal and nicotine-induced reinstatement.	Murine model: a nicotine-conditioned place preference.	Naive male 8- to 10-week-old ICR mice.	CEF (200 mg/kg, twice per day) for 4 consecutive days.	CEF did not disrupt the acquisition of nicotine CPP. CEF blocked nicotine-primed reinstatement of nicotine CPP. Physical and affective precipitated nicotine withdrawal signs are attenuated by CEF. Repeated CEF failed to enhance the low dose of nicotine (0.5 mg/kg) in the tail flick or the hot plate assays or body temperature assessments.	Alajaji et al., 2013
Cocaine-seeking relapse.	Murine model: cocaine self-administration.	Rats.	CEF (200 mg/kg IP) for 7 days.	CEF restored GLT-1 and xCT levels and prevented cue- and cocaine-induced reinstatement of drug-seeking behavior.	Knackstedt et al., 2011
Cannabinoid tolerance	Murine model: cannabinoid tolerance.	Male Balb-c albino mice.	CEF, with its higher doses (100–200 mg/kg),	Attenuated the development of tolerance to the analgesic and hypothermic. Had no effect on its cataleptic action. Upregulated the GLT-1 expression.	Gunduz et al., 2011b
Morphine-induced dependence and Tolerance.	Murine model: morphine-induced dependence and Tolerance.	Adult male Albino mice.	CEF (50, 100, and 200 mg/kg).	Attenuated the development of tolerance to the antinociceptive effect. Reduced naloxone-precipitated withdrawal jumping and standing on feet.	Habibi-Asl et al., 2014

*CEF, ceftriaxone; GLT 1, glutamate transporter 1; NAc, nucleus accumbes pars compacta; EAAT 2, excitatory amino acid transporter 2; i.p., intraperitoneally*.

Therefore, this review is intended to describe the pharmacological effects of CEF in various CNS related disorders conducted in preclinical and clinical studies along with its respective suggested neuroprotective mechanisms.

## Effects of CEF Against Neurodegenerative Disorders

Neurodegenerative diseases are a diverse set of disorders characterized by a progressive loss of neuronal structure and function in distinct sections of the central nervous system. The neurological sequel of neurodegeneration results in a devastating outcome on mental as well as physical functioning of patients (Gao and Hong, 2008; Cannon and Greenamyre, 2011). Among many neurodegenerative disorders, Alzheimer's disease (AD) and Parkinson's disease (PD) are the most commonly encountered disorders (Xie et al., 2014). Huntington's disease (HD) and amyotrophic lateral sclerosis (ALS) are still devastating neurological disorders, though less prevalent (Siddique and Siddique, 2008; Franco-Iborra et al., 2018). To date, there is no cure or disease-modifying agents available for most of these neurodegenerative disorders and therapy is currently focused on symptomatic management and supportive care so as to improve the quality of life of the patients (Xie et al., 2014; Cummings, 2017).

**Figure 1:**
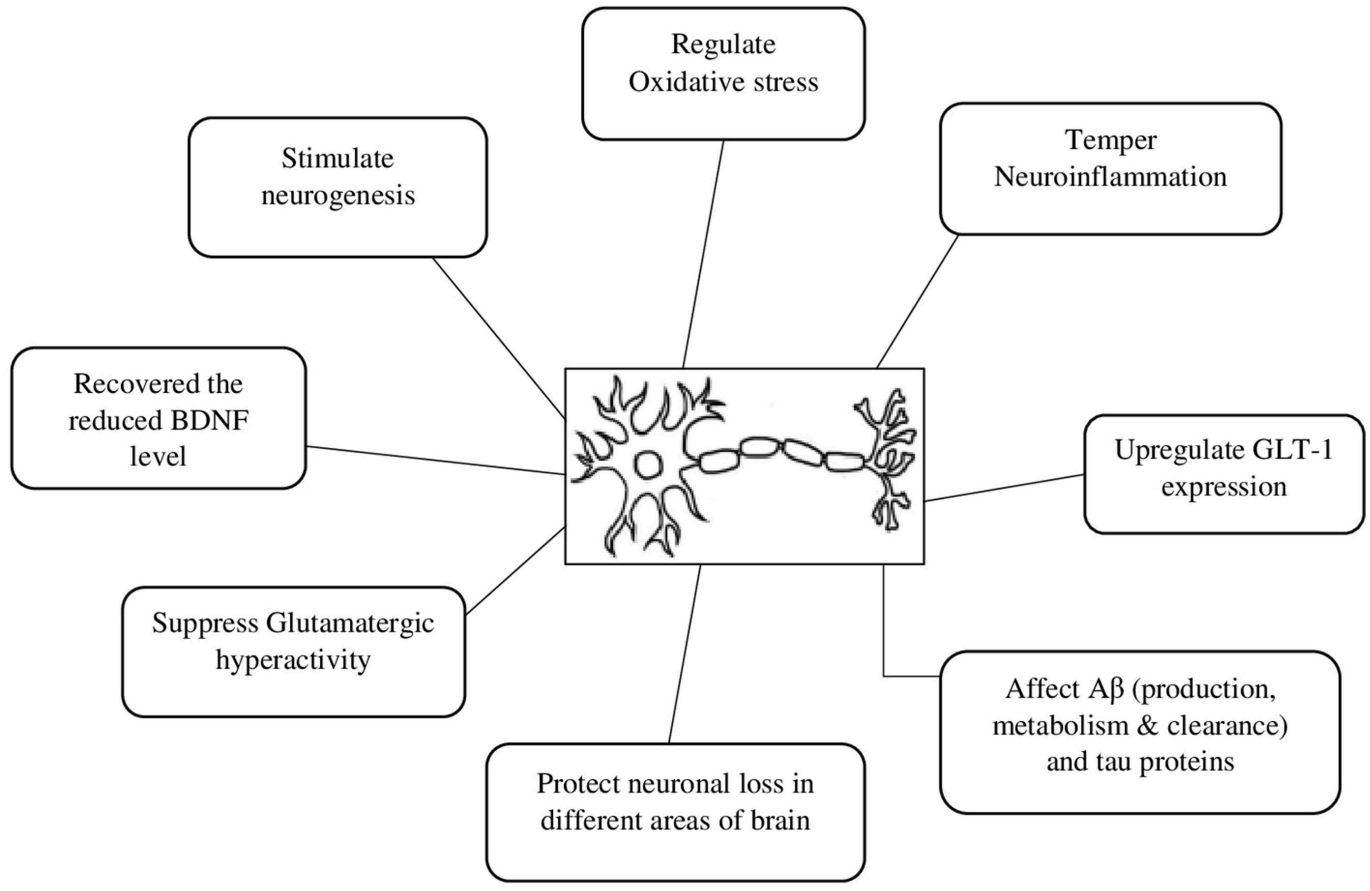
Possible neuroprotective mechanisms of ceftriaxone. Aβ, beta amyloid protein; BDNF, brain-derived neurotrophic factor; GLT 1, Glutamate transporter 1.

A beta-lactam antibiotic, CEF is currently attracting the scientific community due to its multiple mechanisms to relieve symptoms and modify the natural history of various neurodegenerative diseases. Of the different possible mechanisms, upregulation of GLT-1 expression, attenuation of oxidative stress, and neuroinflammation are among the common suggested mechanisms for its neuroprotective actions (Table 1 and Figure 1). For instance, in animal models of Parkinson's disease, CEF exhibited a recovery of memory deficits (Huang et al., 2015), ameliorated abnormal uncontrolled movements (Chotibut et al., 2017), attenuated oxidative damage and restored the reduced levels of endogenous antioxidant enzymes (Bisht et al., 2014; Kaur and Prakash, 2017). Additionally, CEF modulated the expression of tyrosine hydroxylase (Chotibut et al., 2014), α-synuclein expression (Ho et al., 2018), and neuroinflammation (Kaur and Prakash, 2017) as well as prevented dopaminergic degeneration (Ho et al., 2014), while upregulating the levels of GLT-1 expression and glutamate uptake (Chotibut et al., 2014).

Alzheimer's disease (AD) is one of the most prevalent forms of dementia (Sadigh-Eteghad et al., 2015). Different animal models of Alzheimer's disease were affected by the administration of CEF (Table 1). Despite the most commonly underlining protein implicated in the pathology of AD, beta-amyloid (Aβ) protein was not directly affected (Zumkehr et al., 2015), CEF downregulated the messenger RNA (mRNA) expression of *Bace1* (a gene that encodes β-secretase involved in Aβ formation), *Ace2* (a gene that encodes enzymes play a part in Aβ metabolism), and the expression of gene of β-actin. Furthermore, CEF amplified the gene expression of *Mme* and *Ide* (genes that encode enzymes involved in Aβ degradation) as well as the expression of *EPO* gene (a gene that encodes erythropoietin correlated to endothelial function and removal of Aβ) (Tikhonova et al., 2018). CEF-treated animals also showed improvements in memory impairments and restoration of cognitive function and neuronal density (Tikhonova et al., 2017). CEF also attenuated increased levels of acetylcholine esterase enzyme and oxidative stress (Akina et al., 2013). In addition, CEF administration to animal models of Alzheimer's disease displayed an upregulation of GLT-1 expression, preservation of synaptic proteins and downregulation of *tau* proteins (Zumkehr et al., 2015). Furthermore, CEF showed neuroprotective actions in various models of amyotrophic lateral sclerosis (Lewerenz et al., 2009; Yamada and Jinno, 2011; Cudkowicz et al., 2014) and Huntington's disease (Miller et al., 2008; Sari et al., 2010).

## Analgesic Effect of CEF

Analgesic effects of the beta-lactam antibiotic CEF, in different preclinical animal models (nociceptive and neuropathic pain models) have also been reported (Table 2). In a neuropathic pain model in rats, CEF-treated groups showed a meaningful overexpression of GLT-1 level and glutamate uptake in the spinal dorsal horn (Hu et al., 2010). Another study on a similar model showed that CEF significantly attenuated the production of TNF-α and IL-1β (Amin et al., 2012). CEF mitigated the increased levels of Bax and cleaved forms of caspases 3 and 9, while the expression of Bcl2 was markedly amplified (Amin et al., 2014). CEF inhibited opioid-induced hyperalgesia (OIH) in mice. Further, the beta-lactam antibiotic, CEF reversed the OIH induced downregulation of GLT-1 expression (Chen et al., 2012). Finally, CEF produced marked dose-dependent antihyperalgesic effects in the somatic inflammatory and visceral pain models (Stepanovic-Petrovic' et al., 2014).

## Protective Effect of CEF Against Brain Ischemia

Various studies reported the protective role of CEF against different models of brain ischemia (Table 2). CEF pre-treatment reduced infarct volume and apoptotic index and CEF treatment significantly increased GLT-1 mRNA and protein levels (Mimura et al., 2011). In a rat model of global brain ischemia, it was found that pre-treatment with CEF substantially prevented the delayed neuronal death in the hippocampal CA1 area commonly induced by global brain ischemia (Hu et al., 2015; Table 2).

## Protective Effect of CEF Against Stroke and Traumatic Brain Injury

CEF also showed protective activity on animal models of stroke (Table 2). CEF strongly reduced infarct size, marked improvement of neuronal survival within the penumbra, and diminished neurological impairment (Thöne-Reineke et al., 2008). Furthermore, CEF was found to produce promising protective effects against different models of brain injury (Cui et al., 2014; Table 2).

## Effect of CEF on Alcohol Dependence and Withdrawal

Studies evidenced that glutamate transmission is implicated in various features of drug addiction (Sari et al., 2011). The foremost transporter of glutamate, GLT1, in the nucleus accumbens (NAc) and prefrontal cortex (PFC) plays a notable role in alcohol dependence behavior attenuation (Rao and Sari, 2014a). CEF is found to be the most effective β-lactam antibiotic in increasing the expression of GLT1 in the brain (Rothstein et al., 2005). Though CEF attenuated ethanol consumption in P rats, only the relatively higher doses (100 and 200 mg/kg) were associated with marked upregulation of GLT1 expression in the PFC and NAc. This increases in the expression of GLT1 appear to be inversely associated with a post treatment attenuation of ethanol intake (Sari et al., 2011). According to Lee et al. CEF induced indirect upregulation of GLT-1 expression and GSH via the activation of NF-κB, specifically at the NF-κB binding site (272 position) of the GLT-1 promoter (Lee et al., 2008). Another study has also revealed that CEF treatment improved GSH and xCT (a catalytic subunit of xC^−^) levels in PFC, NAc, and associated brain regions (Lewerenz et al., 2009). This CEF-induced increase in the xCT level was found to be correlated with downregulation of extracellular levels of glutamate (Sari et al., 2011), which is the very reason for alcohol dependence. On the other hand, upregulation in the levels of xCT and subsequent upsurges of GSH level may be one possible way to reverse the GLT impairments caused by free radical overproduction. Ethanol withdrawal is related to an increment of oxygen-derived free radicals (Labra Ruiz et al., 2018), which in turn have been shown to inhibit glutamate uptake by oxidation of thiol groups (Volterra et al., 1994).

During CEF treatment in ethanol dependence, an increase in water intake was observed that could be due to the compensatory effect of water to the decrease in ethanol intake (Sari et al., 2011; Table 3). On the other side, GLT1 and ENT1 were inversely affected as a consequence of ethanol consumption and this suggests that the neuroadaptative mechanisms are involving these proteins in both NAc core and shell (Sari et al., 2013).

Alcohol withdrawal syndrome is a medical emergency which is related to significant mortality rates (Campos et al., 2011). CEF administration was found to reduce or almost completely abolish all manifestations of ethanol withdrawal in P and Wistar rat variants and prevented withdrawal-induced escalation of alcohol intake. CEF treatment was also associated with long-term upregulation of excitatory amino acid transporter subtype 2 (EAAT2) in the striatum that was downregulated by ethanol withdrawal (Abulseoud et al., 2014). CEF might therefore be used as a potential therapeutic treatment for the attenuation of relapse-like ethanol-drinking behavior (Alajaji et al., 2013; Qrunfleh et al., 2013; Table 3).

## Effect of CEF on Nicotine Withdrawal and Nicotine-Induced Reinstatement

Tobacco use is the foremost cause of premature mortality in the United States and around the globe. Nicotine is the primarily responsible component for addiction and related behaviors (Castane et al., 2005). Different studies suggested that nicotine-induced adaptations in glutamatergic neurotransmission play an important role in the development of nicotine dependence (Liechti and Markou, 2008). With this regard, CEF administration reversed nicotine withdrawal manifestations and mitigated nicotine-primed reinstatement of nicotine-conditioned place preference (CPP) without affecting acquisition (Alajaji et al., 2013; Table 3).

## Effect of CEF on Acute Cocaine-Evoked Dopaminergic Neurotransmission

CEF exerts its prominent effects on the dopaminergic system in addition to its effects on the glutamatergic system. It decreased the negative effects of cocaine on locomotor and dopamine-activities. This effect was not responsive to a glutamate re-uptake blocker but rather affected some of the principal regulatory components of dopamine transmission in the NAc (Barr et al., 2015; Table 3).

## Effect of CEF on Cannabinoid Tolerance

CEF prevented the development of tolerance to the analgesic and hypothermic actions of WIN 55,212-2 (cannabinoid receptor agonist) by increasing glutamate uptake. Even though the mechanism is not clear, stimulation of GLT-1 by CEF is one of the suggested mechanisms in preventing the development of tolerance to cannabinoids (Gunduz et al., 2011b; Table 3).

## Effect of CEF on Morphine-Induced Dependence

Morphine-induced tolerance and dependence were diminished when mice were pretreated with amitriptyline and CEF alone or in combination. Moreover, the co-administration of low doses of CEF (50 mg/kg) and amitriptyline (5 mg/kg) significantly attenuated morphine-induced dependence compared to the administration of CEF or amitriptyline alone. This enhanced effect could be due to the additive or synergistic effect of CEF and amitriptyline on morphine-induced drug dependence (Habibi-Asl et al., 2014; Table 3).

## Antidepressant Effects of CEF

For a long time, depressive disorder (DD) was thought to be attributed mainly to biochemical alterations of the monoamines and their receptors (Labra Ruiz et al., 2018). A majority of the currently available antidepressants were developed based on this hypothesis and act by increasing the availability of the monoamines including norepinephrine, dopamine, and serotonin. This is effected either by preventing the metabolism of these neurotransmitters or by blocking the transporter mediated reuptake of the these neurotransmitters (Delgado, 2000; Fiedorowicz and Swartz, 2004).

Moreover, DD is thought to be related to excessive glutamatergic neurotransmission. In such cases increased extracellular concentrations of glutamate were observed in several brain regions (Lowy et al., 2002). In another way, GLT1 expression is elevated as a compensatory mechanism to overcome the increased stress-induced glutamate release (Delgado, 2000). Recent evidence revealed that the β-lactam antibiotic, CEF enhanced the uptake of glutamate via the up-regulation of GLT1, suggesting its antidepressant effect. CEF treated animals also showed a marked decrement of immobility in the forced swim and tail suspension tests. Even though not statistically significant, a similar trend was noted in novelty-suppressed feeding (Mineur et al., 2007; Borah et al., 2018). A more recent study also showed that the antidepressant effect of CEF was comparable to that of fluoxetine in the tail suspension test in a dose-dependent manner (Borah et al., 2018).

## Effects of CEF in Seizure Modulation

It was assumed that oxidative stress and reactive oxygen species (ROS) produced an important role in the progression of epileptic-seizures because they gradually disrupt the intracellular calcium homeostasis, which leads to neuronal loss (Chang and Yu, 2010). A study revealed that pre-treatment of animals with CEF provided meaningful protective actions against pentylenetetrazole (PTZ)-induced generalized clonic seizure, generalized tonic-clonic seizure, and convulsion-associated deaths (Jelenkovic et al., 2008).

A similar study also showed that CEF treatment substantially improved PTZ-induced convulsions and caused a noticeable modulation of oxidative stress indicators and Connexin 43 expression in the CA3 region of the hippocampal area. CEF also prominently reduced tonic-clonic convulsions and duration of these convulsions and prolonged the latency time in the PTZ-kindling model (Hussein et al., 2016).

## The Possible Mechanism of Actions of CEF in Neurological Disorders

After induction of different models of neurological illness by lesioning or chemicals, it is known that animals manifest behavioral alterations in early onset and in the long-term they might also exhibit memory deficits (Van Dam and De Deyn, 2011; Savio et al., 2012; More et al., 2016). Numerous studies evidenced that CEF substantially improved such chemical-induced behavioral alteration and memory impairments in different rodent models of neurological conditions (Lai et al., 2011; Wei et al., 2012; Akina et al., 2013; Chotibut et al., 2014; Feng et al., 2014; Hsieh et al., 2017; Kaur and Prakash, 2017; Fan et al., 2018).

It is well-known that an abnormally increased level of glutamate (Glu) in the brain can induce neuronal damage and excitotoxicity that contributes to the pathogenesis of various neurological disorders including epilepsy, ALS, cerebral ischemia, parkinsonism, and AD (Massie et al., 2010; Annweiler et al., 2014; Kleteckova et al., 2014). There are different glutamate transporters involved in terminating glutamatergic transmission and physiological actions (Kanai et al., 2013; Divito and Underhill, 2014). The pre-synaptic glutamate transporter, GLT-1, clears most of the glutamate released in the cortex and hippocampus (Scofield and Kalivas, 2014). Furthermore, GLT-1 is one of the most common transporters and consists of about 80% of the glutamate transporters expressed in the hippocampus (Mookherjee et al., 2011). There is also evolving evidence suggesting a blockade of certain Glu receptors and/ or enhancing the expression of GLT-1 shown to improve neurological outcomes in various experimental models of neurological illnesses (Zlotnik et al., 2008, 2009; Wang et al., 2014; Bai et al., 2016). Among different agents tested, CEF is one of the beta-lactam antibiotics reported to have neuroprotective actions.

Several studies have revealed that the mechanism of action behind the beneficial effect of CEF in neurodegenerative diseases like PD (Leung et al., 2012; Chotibut et al., 2014, 2017; Hsu et al., 2015; Zhang et al., 2015), AD (Zumkehr et al., 2015), HD (Miller et al., 2008; Sari et al., 2010), and ALS (Yamada and Jinno, 2011) is mediated by enhancing the expression of GLT-1 mRNA levels. Similarly, the mechanism behind CEF's analgesic potential has been associated with the upregulation of the pre-synaptic GLT expression (Ramos et al., 2010; Gunduz et al., 2011a; Chen et al., 2012; Macaluso et al., 2013; Nicholson et al., 2014).

The upregulation of GLT-1 expression is associated with the significant attenuation of the neuronal damage caused by brain ischemia, and the overexpression of GLT-1 in the ischemic cortex is associated with a reduction of the size of the lesion and improves behavioral and cognitive recovery (Harvey et al., 2011). Studies also showed the neuroprotective actions of CEF in different models of ischemia (Lai et al., 2011; Mimura et al., 2011; Inui et al., 2013; Hu et al., 2015; Krzyzanowska et al., 2016), TBI (Goodrich et al., 2013; Cui et al., 2014; Feng et al., 2014), and stroke (Lipski et al., 2007; Thöne-Reineke et al., 2008) supposedly via an increased expression of GLT-1.

Abnormal regulation of glutamatergic neurotransmission, due to an excessive amount of Glu in brain reward circuitry, has been involved in both initiation and expression of addiction to the drug of abuse related behavior (Kalivas et al., 2009; D'Souza, 2015; Liu et al., 2017). It has also been demonstrated that the expression of the catalytic subunit of xC^−^, xCT, and GLT-1 are reduced in the NAc following substance of abuse (Massie et al., 2015; Roberts-Wolfe and Kalivas, 2015). From various preclinical studies, it has been reported that CEF markedly attenuates alcohol/ drug-seeking behavior and drug clued-up recall while restoring the reduced levels of both xCT as well as GLT-1 (Knackstedt et al., 2011; Sari et al., 2011, 2013; Qrunfleh et al., 2013; Abulseoud et al., 2014).

Long-lasting neuroinflammation and oxidative stress are the other pathological processes involved in brain aging and neurodegeneration (Lee et al., 2008; Mishra et al., 2016; Shah et al., 2016). It has been reported that neuroinflammation is a key player in various neurological disorders, including neurodegenerative illnesses and CNS injury (Chen et al., 2016; Yang and Zhou, 2018). Hence, controlling of the neuroinflammation and excitotoxicity are supposed to limit the abnormal alterations and progression of different neurological disorders (Zhou and Hu, 2013; Yimer et al., 2019), which is further supported by evidence reported, where the use of anti-inflammatory agents or targeting inflammatory markers prominently prevented or at least reduced the progression of neurological disorders (Gagne and Power, 2010; Decourt et al., 2016). Fortunately, pre-treatment of CEF showed attenuation of proinflammatory mediators including NF-κB, IL-1β, INF-γ, and/ or TNF-α in various models of neurological disorders such as PD (Kaur and Prakash, 2017), TBI (Wei et al., 2012), neuropathic pain (Pottabathini et al., 2016), and cerebral ischemia (Lujia et al., 2014), which might contribute its own share of reported neuroprotective actions of CEF.

In addition to neuroinflammation, oxidative stress, and mitochondrial dysfunction have also been involved in the development and progression of a wide range of neurodegenerative and mental disorders (Tobe, 2013; Rossignol and Frye, 2014; Yimer et al., 2019). There were also some reports suggesting that antioxidant therapy moderated the production of reactive oxygen species and prevented downstream pathologies of certain neurological diseases (Uttara et al., 2009; Du et al., 2017). Similarly, there are reports that animal models of different neurological disorder received CEF, which attenuated oxidative stress by either reducing oxidative markers such as MDA or by improving the endogenous antioxidant actions such as GSH and SOD (Lewerenz et al., 2009; Altaş et al., 2013).

BDNF is a neurotrophic factor which plays a crucial role in neuronal survival, neurogenesis, and plasticity. It has also been reported that abnormal BDNF expression is involved in several neurological illnesses. There are few studies that report CEF caused upregulation and restoration of reduced BDNF levels in certain animal models (Lee et al., 2008; Kaur and Prakash, 2017).

Besides, CEF might exert its neuroprotective actions through other mechanisms such as by affecting Aβ and tau protein metabolism and clearance in an AD model, prevent polymerization of α-synuclein in DLB (Ho et al., 2018) and PD (Ruzza et al., 2014; Tikhonova et al., 2018) models, which is of course, a call for further research to substantiate that the neuroprotective actions of CEF is also mediated via these important pathological proteins.

Generally, it appears that the shared mechanism of actions of CEF in the range of various neurologic disorders is through the upregulation of GLT-1 expression and the reduction of proinflammatory mediators and oxidative stress. However, this requires further investigation in order to verify the suggested and possible other newer neuroprotective mechanisms of CEF for each neurological ailment.

## Conclusion

This review revealed that the beta-lactam antibiotic, CEF might have neuroprotective actions, which can affect a wide array of neurological disorders including AD, PD, HD, stroke, brain ischemia, seizure, and drug/alcohol dependency and withdrawal. It is particularly interesting as CEF affects the wider pathological states of CNS disorders including glutamatergic system, oxidative stress, neuroinflammation, apoptotic index, and various toxic protein aggregations. Since most of the studies conducted so far are preclinical studies, clinical studies such as randomized clinical trials and further mechanistic studies are required to assure its neuroprotective actions in real clinical scenarios.

## Author Contributions

EY developed the research concept and took initiatives of the work by drafting the manuscript while KT and HH provided greater contributions toward collecting, extracting, and organizing relevant data and also revising the review paper and agreed to be accountable for all aspects of the work.

### Conflict of Interest Statement

The authors declare that the research was conducted in the absence of any commercial or financial relationships that could be construed as a potential conflict of interest.

## Abbreviations

CEF: Ceftriaxone
AD: Alzheimer's disease
HD: Huntington's disease
PD: Parkinson's disease
CNS: central nervous system
TBI: Traumatic brain injury
GLT 1: Glutamate transporter 1
NAc: Nucleus accumbens
PFC: Prefrontal cortex
EAAT2: excitatory amino acid transporter subtype 2
